# A Pulsed Current Application to the Deformation Processing of Materials

**DOI:** 10.3390/ma16186270

**Published:** 2023-09-19

**Authors:** Vladimir Stolyarov, Anna Misochenko

**Affiliations:** Mechanical Engineering Research Institute of Russian Academy of Sciences, 101990 Moscow, Russia; vlstol@mail.ru

**Keywords:** pulsed current, deformation, electroplastic effect, deformation behavior, microstructure, properties, mechanisms of electroplasticity, modeling

## Abstract

A review of studies on the electroplastic effect on the deformation process in various conductive materials and alloys for the last decade has been carried out. Aspects, such as the mode and regimes of electric current, the practical methods of its introduction into materials with different deformation schemes, features of deformation behavior accompanied by a pulsed current of different materials, structural changes caused by the combined action of deformation and current, the influence of structural features on the electroplastic effect, changes in the physical, mechanical, and technological properties of materials subjected to plastic deformation under current, possible mechanisms and methods of physical and computer modeling of the electroplastic effect, and potential and practical applications of the electroplastic effect are considered. The growing research interest in the manifestation of the electroplastic effect in such new modern materials as shape-memory alloys and ultrafine-grained and nanostructured alloys is shown. Various methods of modeling the mechanisms of electroplasticity, especially at the microlevel, are becoming the most realistic approach for the prediction of the deformation behavior and physical and mechanical properties of various materials. Original examples of the practical application of electropulse methods in the processes of drawing, microstamping, and others are given.

## 1. Introduction

The physical phenomena that occur when a pulsed current is applied to solid metal materials are well known. These include the thermal effect, the appearance of a magnetic field (and, as a result, skin and pinch effects), vibration, etc. [1]. Often, such phenomena occur simultaneously. The most notable among the listed effects is the thermal effect, which is often used in electric pulse treatment (EPT) technologies to change various properties due to the structural changes associated with the effect [2,3]. Note that EPT usually refers to technologies in which the object is not subjected to external deformation treatments. In the case of a combined effect on the material of simultaneously pulsed current and plastic deformation (for example, rolling, drawing, pressing, bending, tension, and compression), such processes are called electrically assisted manufacturing (EAM) or electroplastic deformation (EPD) in contrast to EPT. Those processes will be discussed in detail in this review.

The EPD is based on all the above mechanisms in interaction with plastic deformation (that is, with the movement of dislocations), which can be enhanced, leading to increased deformability and carried out at lower stresses due to the occurrence of the electroplastic effect (EPE) [4]. EPE is a phenomenon that depends on many external and internal factors [5]. The external factors include those associated with deformation. They are the scheme of the stressed strain state, strain rate, strain temperature, pulse-current modes, and regimes. Factors, such as the material, its structure, and phase composition, are internal factors.

Unlike many other reviews [6,7] related to one of the areas in the field of EPE, this review presents several important aspects in a comprehensive manner, allowing for an overview of the main problems in the study and application of EPE. 

External and internal factors affecting the deformation behavior and mechanical properties of various materials, the effect of EPD on the microstructure and properties and vice versa, and the effect of microstructure features on EPE are considered in this discussion. In particular, attention is paid to the effect of grain size in the nanoscale, dynamic recrystallization [8], crystallographic texture [9], effect on dislocation density [10], phase transformations [11], and aging effects [12]. 

Information on modeling and the most accepted EPE mechanisms is given [6,13]. The importance of the transition of modeling from the macrolevel to the micro- and atomic scale with the use of molecular dynamics methods and ideas about new defects in the crystal lattice is emphasized. 

Examples are given on the application of pulsed electric current to traditional metal forming processes such as rolling, drawing, stamping, and dimensional processing [14], as well as to new technologies of sintering [15], friction welding, pressure welding, additive technologies, and microforming. 

The benefit from the use of EPE in this area is primarily associated with an increase in deformability [16], a decrease in the applied forces and temperature of deformation (from high to moderate and even room temperature), and the possibility of combining deformation and heating. In some cases, an increase in functional properties associated with an improvement in the uniformity of the structure can be observed.

Overall, the review considers external and internal factors affecting the deformation behavior, microstructure, and properties of different materials. Information on modeling, the most accepted EPE mechanisms, and application examples are also provided. 

## 2. Methods of Current Introduction

For the study or application of EPE, it is important to understand and regulate the influence of many external factors. Such factors should include the source (generator) of current pulses, the current mode and current regimes, and methods of current supply.

Today, three main modes of electric current are known—direct, alternating, and pulsed. Below, we will consider the factors related to pulse current, since the first two modes are less effective for the practical application of EPE. A feature of the pulse current is the variety of adjustable parameters. These include direction, density, frequency, duration, duty cycle, and pulse shape. The passage of pulsed current is accompanied by various phenomena: thermal effect, electro- and magnetoplastic effects, electric and magnetic fields, and vibration. These phenomena are well studied and find practical applications. One of them, namely EPE, is considered an alternative to heat treatment, as well as deformation methods of structure materials [17].

**Figure 1:**
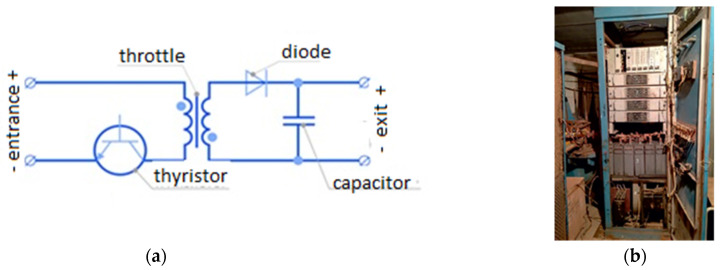
Pulse-current generator: (**a**) electrical scheme; (**b**) appearance [18].

**Figure 2:**
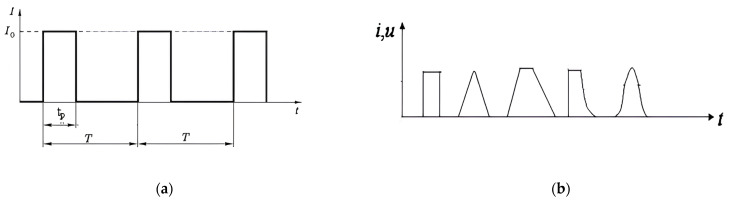
Pulse-current parameters (**a**) and possible pulse forms (**b**).

The source of the pulse current is a generator on a thyristor converter [18], the schematic diagram and appearance of which are shown in Figure 1. The most important technical characteristics of the generator are the value of the maximum output current I_o_, the period T (or the frequency ν), and the minimum pulse duration t_p_ (Figure 2). Various pulse forms are shown in Figure 2b. Currently, known commercial generators with a power of <60 kW have an output of I_o_ = 1–5 kA, a frequency of 1–1000 Hz, and a pulse duration of t_p_ = 1–100,000 μs at a voltage of 10–15 V. [Montecchio Maggiore, Vicenza, Italy; LLC NPCC “Cursor” RF 142184 Klimovsk-4 Moscow region] [19,20]. 

Another parameter of the pulse current is the duty cycle Q = T/t_p_ = 1/ (υ × t_p_) (T is the period, s; υ is the pulse frequency, Hz; t_p_ is the pulse duration, s). A multiple increase in the duty cycle Q >> 1 actually means the transition of the pulse-current mode to the single pulse mode with minimal thermal effect of the current or even the absence of it. Accordingly, a decrease in the duty cycle up to Q = 1 means the action of a multi-pulse current, which should contribute to an increase in the deformation temperature from room to high. 

Studies on the effect of pulse-current duty cycle on EPE are extremely rare and are devoted to applied issues of application to high-speed rolling [20] or to the EPE dependence under tension in coarse-grained (CG) and ultrafine-grained (UFG) titanium [21,22]. For a wide range of duty cycles, it was shown that pulsed current during the tension of CG titanium can lead not only to traditional softening but also to hardening [21]. However, with a low-duty cycle in both types of titanium, only a decrease in flow stresses occurred [22]. 

**Figure 3:**
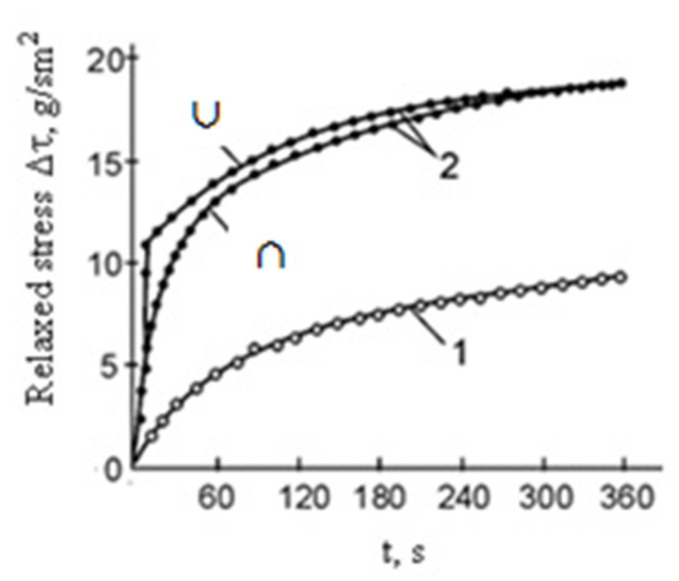
Time dependence of stress relaxation in a Zn single crystal: 1—without current; 2—with current of different directions (∩—unipolar pulses, ∪—bipolar pulses) [20].

The direction and polarity of the current are also considered important factors. It is known from practice that the direction of the pulse current can coincide, be opposite to the direction of deformation (rolling and drawing), or even be oriented at an angle (bending and stamping) [23]. It is assumed that the EPE is maximal when the electron drift velocity coincides with the direction of the external force. In addition, the influence of polarity on the EPE was demonstrated, which indicates the presence of the “electron wind”. In this case, the EPE dependence was linear, not quadratic, for the thermal effect [20,24,25]. This was especially well manifested in relaxation experiments on a Zn single crystal with a size of ∅1 × 30 mm^2^ using tension at a temperature of 78 K and a current density of 400 A/mm^2^ (Figure 3). It is also noted that unipolar pulses have a stronger effect on deformable crystals than bipolar pulses with the same total pulse area, and, accordingly, the same thermal effect [20]. 

It is necessary to note an important requirement for the maximum current produced by the pulse generator. The analysis of articles on the study of EPE in various materials shows that the minimum current density at which there is a noticeable decrease in deformation forces depends on the alloy’s nature and its electrical resistivity. This leads to the concept of a critical (threshold) current density j_cr_ when EPE occurs [26]. It is about 10–500 A/mm^2^ in materials with high resistivity and more than 1000 A/mm^2^ in materials with low electrical resistance (copper and aluminum). As a result, for example, for copper billets with a cross-section of about 10 mm^2^ a generator producing a current of at least I = 10,000 A will be required. Consequently, there is a limit to the use of pulsed current. Hence, electroplastic rolling will be possible only in semifinished products of thin cross sections.

**Figure 4:**
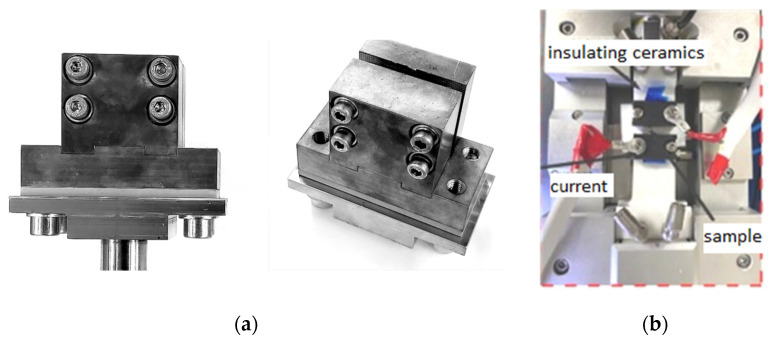
An example of clamping devices for tensile tests made of (**a**) copper [19]; (**b**) ceramics [27].

**Figure 5:**
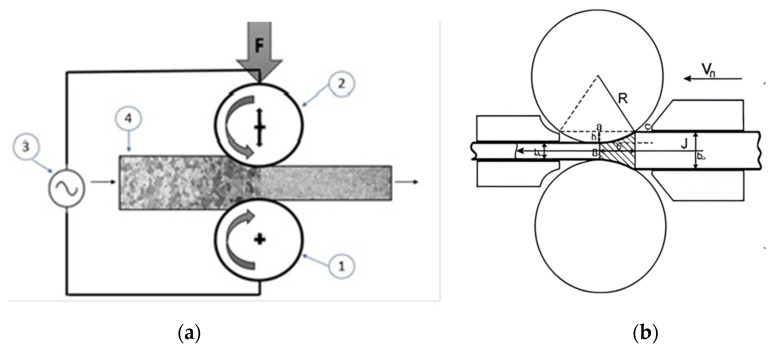
The current supply scheme for rolling: (**a**) from roll 1 to roll 2; 3—a pulse generator; 4—a sample; (**b**) sliding contacts [20].

Let us consider the features of introducing current into a material, which depends on the purpose of the study or practical application. In the case of studying the behavior of materials in the processes, such as tension, compression, bending, etc., special clamps (or devices) are used to avoid sparking. As a rule, they are made of copper [19] or ceramics (Figure 4) [27]. If the use of current during rolling (or drawing) processes is considered, then the schemes shown in Figure 5 are used. In this case, the current is supplied from roll to roll (Figure 5a) [28] or using sliding contacts (Figure 5b) [20].

The modes and regimes of the pulsed current considered above, as well as the methods of its introduction into the workpieces, are the most important EPD parameters. They regulate the ratio of the contributions of various mechanisms and, accordingly, the technological efficiency, structure, and operational properties of deformable materials.

## 3. Deformation Behavior 

**Figure 6:**
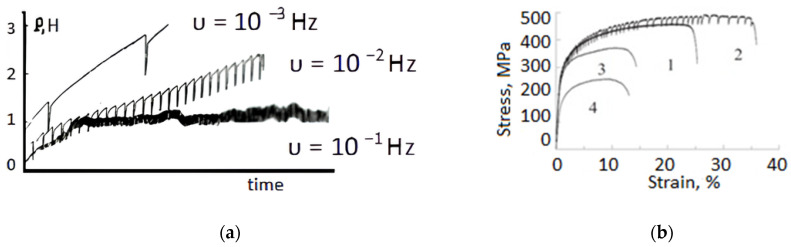
Loading curves by tension of single crystal Zn (**a**) [20] and polycrystal Ti (**b**) [5]: 1—without current; 2—single pulses j = 80 A/mm^2^; 3—multipulse current j = 40 A/mm^2^; 4—direct current j = 6 A/mm^2^.

In contrast with the usual mechanical properties of materials subjected to EPD, the behavior in the process of deformation accompanied by a pulsed current is of particular interest. It is associated with the possibility of visual in situ observation of the loading curve and a deeper understanding of the current effects. The traditional method is tension, which allows adjustment of the parameters for both deformation and the current. The first experiments were performed by the tension of Zn single crystals with single-current pulses. At the same time, downward stress jumps were observed, both in the elastic and elastic–plastic deformation region (Figure 6a) [20]. An increase in the pulse frequency at the same rate of tension led to a decrease in the amplitude of a single jump, the total force, and the coefficient of deformation hardening, which the authors explained by the depletion of the dislocation structure created by active loading of the crystal.

Single-current pulses, in contrast with multipulse and direct current, are capable of causing the effects of strengthening and increasing plasticity, which are clearly visible for an hcp titanium polycrystal (Figure 6b) [5]. A change in the current mode from single pulses to multipulse/direct current sharply reduces the plasticity of polycrystalline titanium. Possible reasons for the decrease in ductility in titanium could be low thermal conductivity and strong neck formation, which contribute to a sharp increase in the current density and, as a consequence, temperature. The hardening effect from a single-current mode is greater, when greater the number of current pulses. Recently, a similar hardening effect at sufficiently low pulsed current densities j = 5 A/mm^2^ was observed in the Ti-7Al alloy, which is uniquely suited for uncoupling Joule heating and EPE [10]. The authors believe that strengthening can be caused by the cross slip of dislocations and twinning. 

**Figure 7:**
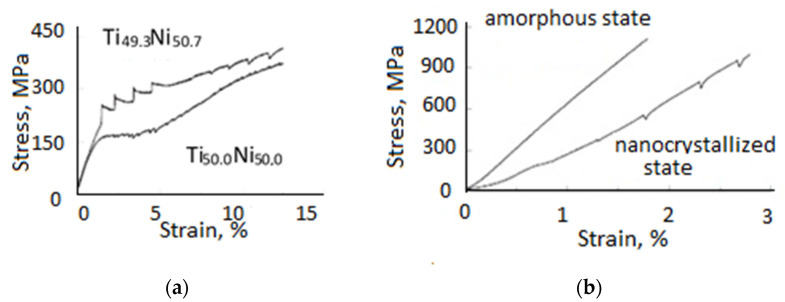
Stress–strain curves of TiNi at current density j = 1500 A/mm^2^ (**a**) and FeSiB at j = 400 A/mm^2^ (**b**) under single-current pulses, t = 1 ms [5].

The original and traditionally investigated EPE in structurally stable metals and alloys manifests itself in the form of downward stress jumps in tensile curves. However, in metastable shape-memory alloys (SMAs) that undergo strain-induced transformations, single-current pulses at different tension stages can lead to the opposite direction of stress jumps. For example, in coarse-grained (CG) TiNi alloys, the sequence of upward and downward jumps changes depending on the phase composition (Figure 7) [5]. In the Ti_49.3_Ni_50.7_ alloy, which is austenitic at room temperature, the stress jumps are directed upward at the pseudo-yielding stage and are caused by the direct A → M transformation. On the contrary, the work-hardening stage showed the downward stress jumps that are directly related to EPE. In the Ti_50_Ni_50_ alloy, which is martensitic at room temperature, the stress jumps are directed downward at the stage of martensite reorientation and are caused by typical EPE. An increase in deformation leads to the reverse M → A transformation and, accordingly, to the shape-memory effect (SME). The difference in the stress jumps amplitude at the same current regime is associated with the morphology of the A and M phases, which are equiaxial and lamellar, respectively.

Most EPE studies are performed on materials in a crystalline state with a large grain size. Due to the differences in amorphous and nanocrystalline materials, as well as their need for deformation treatment, the effect of structural state and grain size on EPE is of interest. In Figure 7b, an example of tension curves by a single pulse current is given for a melting FeSiB spun film in the amorphous and nanocrystallized state with a grain size of 10 nm achieved by annealing at 700 °C for 10 ms. Even though the thermal effect is 3–4 times greater in the amorphous state (due to the higher electrical resistivity [29]), EPE appears only in crystalline film. This is consistent with the proposed EPE mechanism of electron wind, which is realized only in the presence of mobile dislocations. It is known that there are no dislocations in the amorphous state. Something similar was observed on an amorphous cobalt-based alloy under current density j = 4 × 103 A/mm^2^. In this case, the authors explained the effect by structure relaxation [30].

Recently, a large number of publications have appeared on the relaxation phenomena of structure and deformation at the moment of stopping the test without current [31,32] or transmission of single-current pulses observed on tensile curves [33]. In almost all cases, relaxation contributed to the improvement of plasticity. 

**Figure 8:**
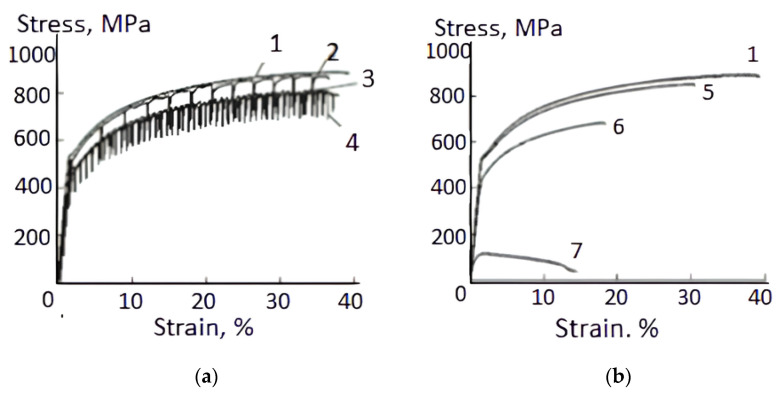
Tensile stress–strain curves under current density 1600 A/mm^2^ (**a**) and 200 A/mm^2^ (**b**) at duty cycle: 1—no current; 2—q = 20,000; 3—q = 12,000; 4—q = 4000; 5—q = 100; 6—q = 20; 7—q = 10 [34].

As mentioned earlier, the pulse-current duty cycle, as one of the important parameters, is emerging in EPE research [21,22,34]. Figure 8 shows the stress–strain curves of aluminum bronze under the current of different duty cycles and densities. They show that a change in the duty cycle in the range of 10–20,000 makes it possible to regulate stress reduction at different current densities [34]. 

As follows from the above examples, the deformation behavior of materials under tension accompanied by a pulsed current can vary from typical, often observed, softening to a weak, but noticeable, hardening. In this case, plasticity, as a rule, decreases due to the formation of a neck. The electroplastic effect exists in the form of the flow stresses or the amplitude of stress jumps decreasing. It rises with an increase in the duty cycle, and achieves a current density above the critical one.

## 4. Microstructure Features

The physical basis of EPD is the interaction of pulsed current (or “electron wind”) with defects in the crystal lattice during the deformation of materials (EPE), as well as the occurrence of concomitant thermal and pinch effects. Therefore, it is of particular interest to study the features of the microstructure under the simultaneous influence of plastic deformation and pulsed current. A comparison of structural features during deformation with current and without current will allow a better understanding of the accompanying effects nature.

The electrical and thermal energy supply usually leads to structural rearrangements, such as a decrease in dislocation density [35], the appearance of twins [36], dynamic recrystallization [37], grain refinement [38], the evolution of crystallographic texture [39], and the formation of oriented microstructures [40,41], as well as the redistribution of inclusions and the effects of aging [42].

**Figure 9:**
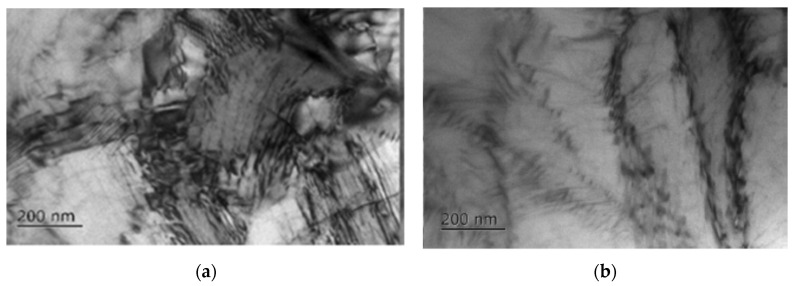
Microstructure of Ti-6Al-4V alloy after bending without current (**a**) and with current (**b**) [45].

The current slows down the deformation processes and acts on dislocations clusters, slowing down the destruction processes and, thereby, increasing the deformability. Dislocations arising during deformation accumulate near the grain boundaries, which is an obstacle to the movement of subsequent dislocations [43]. The energy impact caused by electric current can weaken the strength of interatomic bonds, affecting the movement and annihilation of some dislocations. Hence, an increase in plasticity occurs when the flow stress decreases during deformation [44]. In [45], there is also a decrease in the dislocation density in the Ti-6Al-4V alloy deformed with current compared to deformation (bending) without current (Figure 9).

**Figure 10:**
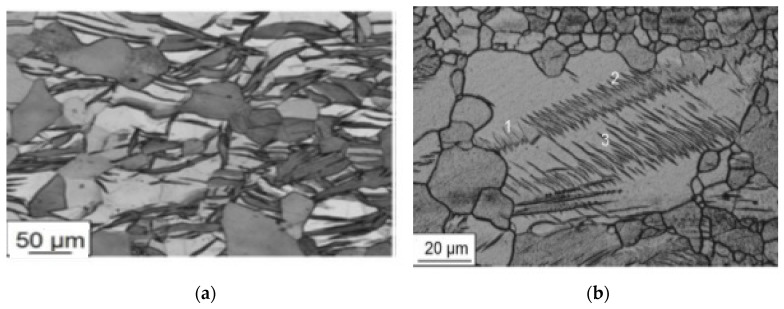
Morphology of the twins formation in the AZ31 alloy under deformation (**a**) and under the combination of deformation and pulse current (1–3 are the different groups of twins) (**b**) [46].

The work [36] describes the formation of twins and dislocation slip as two competing mechanisms during deformation with applied current. Which of the mechanisms prevails largely depends on the used parameters, including the frequency of electrical pulses. It is shown in [46] that an essential criterion responsible for the difference between the twins resulting from deformation and the additional application of electric current is the place of nucleation of the twins and the location (distribution pattern) of the twins in the grain volume. When only mechanical load is introduced, the twins grow primarily at the grain boundaries, forming coarse twins (Figure 10a) and insignificant parts of small twins. When an electric current is applied, strips of thin and small twins are formed inside the grain volume (Figure 10b).

Studies of crystallographic texture show that electropulse action during plastic deformation stimulates the process of recrystallization and twinning with a change in the orientation of fiber distribution compared to other heat treatments [47].

**Figure 11:**
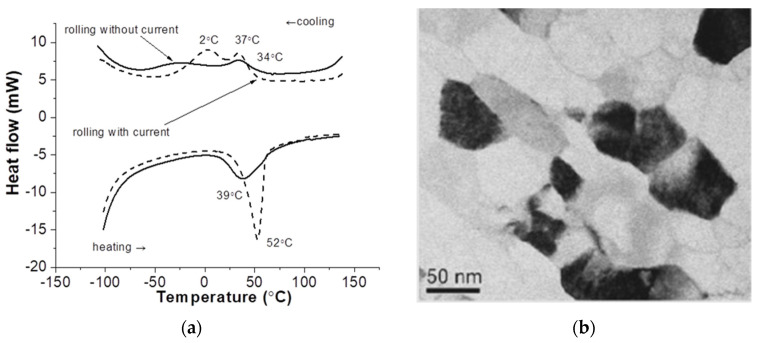
Martensitic transformations after rolling without current and with current (**a**) and microstructure after annealing at 450 °C in Ti_50.0_ Ni_50.0_ alloy (**b**) [51].

A number of studies show that electrical current can contribute to dynamic recrystallization during deformation [48]. In [28] the structure of pure titanium was observed during rolling with current and recrystallized grains with an average size of 8–12 μm were found after such treatment. Recrystallization can be controlled by reduction and current force in order to obtain the necessary grain sizes without postdeformation annealing operations. Studies on a TiNi-based shape-memory alloy show the possibility of creating a nanostructured state by varying the recrystallization temperature [49] and controlling phase transformations when using current during rolling deformation [50]. In particular, the effect of rolling with current on the temperatures of martensitic transformations is shown. The relaxation mechanism of the current action is noted, which consists of a lower intensity of deformation processes compared to cold rolling without current. For example, it is shown that while the cold rolling of a TiNi alloy to the level of true strain e = 0.7 leads to a suppression of the martensitic transformation, rolling with current to the same level of deformation allows the martensitic transformation to take place (Figure 11a) [51]. At the same time, postdeformation annealing leads to the formation of a nanoscale structure (40–50 nm) (Figure 11b).

The structure refinement effect, when using a combination of rolling and current, was also shown in pure zirconium [52] and brass [53]. It has been observed that the impact of the pulse current leads to recrystallization with subsequent grain growth. The average grain size in the recrystallized material was 0.5 μm, which indicates that cryogenic rolling in combination with current is suitable for obtaining the ultrafine-grain microstructure of Cu-30Zn brass.

**Figure 12:**
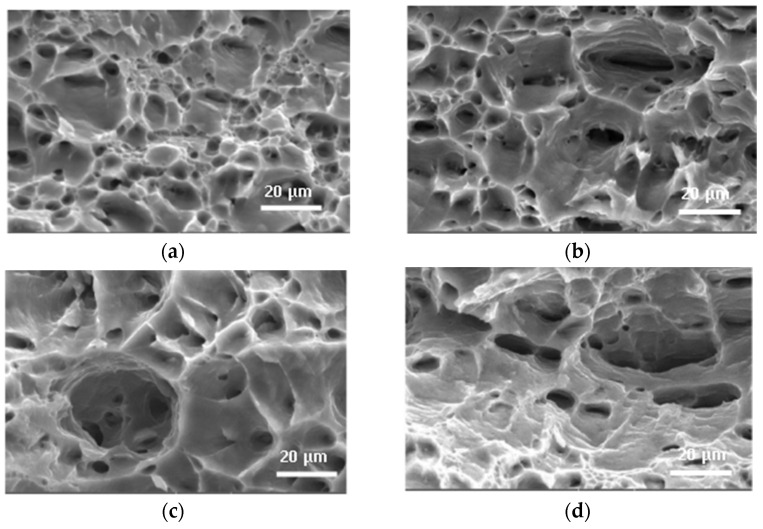
The SEM images of fracture surface under the tension of 6016 aluminum-based alloy without current (**a**,**c**) and with current (**b**,**d**) in the state after natural aging (**a**,**b**) and quenching (**c**,**d**) [42].

A number of papers are related to the study of the electrical current effect in the deformation process on the dissolution of secondary phases or particle formation and aging. Thus, paper [54] shows the possibility of additional structure refinement due to the particle formation. Work [42] describes the structural changes in 6016 aluminum alloy in various initial states (quenched, annealed, and naturally aged) during tension with and without current. The authors noted brittle intergranular fracture in all states; however, they observed some differences in fracture for specimens under tension with and without current (Figure 12). After applying the current, intergranular delamination occurs less frequently and the discontinuous edges become longer. There is also a decrease in the number of particles of the second phases when applying current or a possibility of their crushing and grinding in the case of tension with current.

The fracture nature of TC11 titanium alloy under tension was investigated by the authors in [55]. It is noted that the surface after tension without current contains a large number of small and shallow dimples, whereas the fracture of the sample during tension with a pulsed current contains large and deep cups, and the size of the cups increases with the increasing current density (from 0 to 15 A/mm^2^).

In addition, the use of pulsed current improves the distribution of macro- and microdefects and, in some cases, can reduce or eliminate these defects in many metals and alloys [56].

Note, that the concepts of “deformation with current—microstructure” are interdependent and affect each other. In particular, the variation of the initial phase composition, grain size, etc. will influence the deformation behavior and related effects. Thus, in [57], the influence of grain size and current density on annealed pure copper in the process of deformation with current is investigated. It has been shown that the effect of stress reduction decreases with increasing grain size. It was demonstrated in [58,59] that a decrease in grain size increases Joule heating and increases the reduction of tensile stresses with the current in brass and Ti-6Al-4V alloy.

It should also be noted that the authors, who do not set a task of heating with electrical current, do not observe significant structural changes, in particular grain growth under the simultaneous influence of current and deformation. That confirms the presence of an athermal component of the electroplastic effect [60].

Thus, as a rule, the observed structural features during deformation with current are local in nature and relate, for example, to small differences in grain misorientation or to the morphology of the formation of twins [46]. This fact leads to an additional research interest but also contributes to complications in understanding and explaining the nature of the electroplastic effect, which, despite the variety of works on this topic, still remains open.

## 5. Physical–Mechanical and Technological Properties

One of the most important technological properties, which is influenced by deformation with current, is deformability. This parameter is primarily of interest to researchers studying EPE. Due to the increase in deformability, pressure treatment becomes possible without increasing the temperatures of brittle materials that are difficult to deform under normal conditions. A number of studies show that deformation with current can achieve better deformability of titanium alloys [61,62], magnesium alloys [16,27,63,64], aluminum [65,66], shape-memory alloys based on TiNi [67,68], and some other materials [69].

A decrease in the flow stress under the pulsed current can lead to an increase in the relative elongation of the material [70]. The paper [28] indicates a decrease in the limiting thickness of the titanium sheet during rolling with current compared to cold rolling, which also depends on the deformation rate, i.e., deformability decreases with increasing speed when other parameters remain unchanged. It was shown in [71] that the difference in elongation can reach 3–5% during tension with a current of low densities (5–10 A/mm^2^) compared to tension without current in AA1050 aluminum alloy. In addition, a number of researchers are studying the increase in deformability with current using nonstandard test schemes, for example, shear deformation of the Ti64 alloy [72]. In [45], an increase in deformability of up to 52% was observed in Ti64 alloy using bending deformation under the influence of a pulsed current.

An increase in strength with a simultaneous increase in plasticity is achieved by a combination of deformation and current, usually due to more intensive refinement of the microstructure. Thus, in [52], an increase in the tensile strength of pure zirconium from 450 to 600 MPa was demonstrated using a combination of rolling with current and subsequent low-temperature annealing. At the same time, the preservation of high plasticity (about 25%) was noted. Combined processing, including rolling with current and aging, was studied in the AZ91 alloy. The tensile strength, yield strength, and elongation to fracture were improved by 11–12%, 10%, and 70–75%, respectively [73].

**Figure 13:**
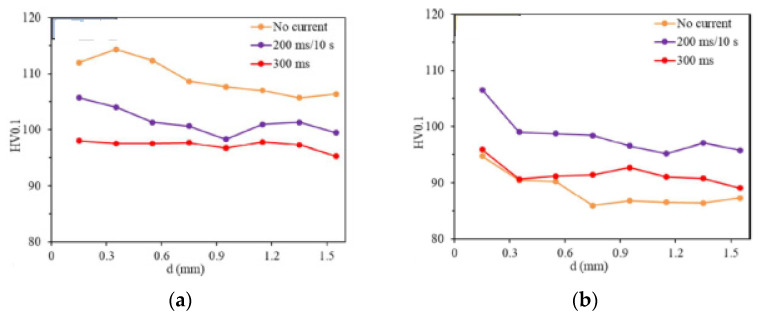
Microhardness of 6016 aluminum alloy after deformation with and without current: (**a**) natural aging; (**b**) quenching (525 °C/water) [42].

However, some researchers note a decrease in plasticity under the action of current, but these effects are found in alloys disposed to aging [42]. The microhardness (Figure 13a) of naturally aged 6016 aluminum alloy was reduced by a combination of rolling and current. The parameter d in Figure 13 is the distance from the edge of the fracture along the rolling direction after a tensile test. However, in the case of the state after quenching from 525 °C in the water, the applied current led to an intensification of the aging process without reducing its plasticity at the selected current regimes, as well as to an increase in strength and microhardness (Figure 13b) [42]. However, strength and ductility decreased for the alloy after natural aging, as shown in this study.

**Figure 14:**
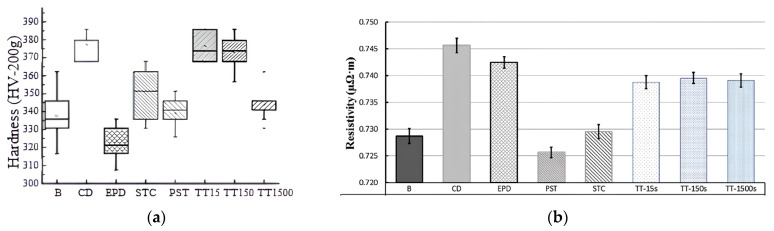
Hardness (**a**) and electrical resistance (**b**) of stainless steel: B—initial undeformed, CD—after drawing, EPD—drawing with simultaneous application of current, STC—drawing with subsequent electric pulse treatment, PST—electric pulse treatment without deformation, TT—annealing after drawing with appropriate duration [47].

The effect of various combinations of current and drawing deformation on the mechanical properties of 308 L stainless steel was investigated in [47]. It is shown that the samples have the highest hardness after drawing without and with short-term annealing. The initial undeformed samples, as well as samples after drawing with subsequent electric pulse treatment, or after standard annealing, demonstrate a lower, but similar, hardness. A simultaneous exposure to current during the drawing process leads to the greatest decrease in hardness (Figure 14a).

The above work [47] also demonstrates the effect of these treatments on electrical resistance (Figure 14b). It can be seen that the resistivity after simultaneous exposure to drawing and current is significantly higher than in the initial material state and is at the level of a deformed material. Note the significant difference in electrical resistance that depends on the sequence of current input: current treatment after deformation significantly reduces this value.

In [45], the authors note a decrease in the friction coefficient under the action of current pulses during deformation by bending of the Ti-6Al-4V alloy. In study [74], a decrease in the adhesion (molecular) component of the friction coefficient, as well as a reduction in gripping during the friction of the TiNi shape-memory alloy after rolling with current, was noted. However, the role of current in this paper is not obvious and the results are related to the structure refinement.

The effect of current on fatigue properties is demonstrated in [75]. It is shown that the introduction of current can significantly increase the number of cycles to failure (from 15,000 to 18,000) for steel.

In a number of papers, the functional properties of shape-memory alloys and their dependence on the introduction of current during deformation were studied [76,77]. There is an increase in reversible deformation, reactive stresses, and superelastic properties compared to rolling without current. In work [78], an increase to 90–96% in the shape restoration coefficient was shown when rolling with a current is performed to e = 1. An increase in the e above one does not have a significant effect. The effect of superelastic behavior after rolling with simultaneous exposure to pulsed current was found in Ti_50.0_ Ni_50.0_ alloy.

Thus, the simultaneous effect of deformation and current can have a significant impact not only on the technological properties (increase in deformability) but also be one of the methods to control the mechanical and functional properties (electrical, shape-memory properties, etc.) in alloys with different natures.

## 6. The Mechanisms and Modeling of EPE

Despite the growing interest in EPE-related problems, the nature of the effect remains completely unexplored, and the proposed mechanisms are theoretical. The formulation of a physical experiment explaining the essence of EPE is difficult (many mechanisms and the complexity of separating the thermal and athermal components). Therefore, a number of papers are related to modeling the accompanying EPE effects. 

A recent review of the current mechanisms of electroplasticity [79] confirms that there is still no unified theory of this phenomenon. Moreover, there are seven confirmed operating mechanisms (the «electronic wind», inertia, thermal fluctuation, magnetoplasticity, hot dislocations, dynamic deformation aging, and absence of EPE) and the same number of previously known phenomena unrelated to EPE (Joule heating, thermal expansion, thermal softening, temperature gradient, pinch effect, magnetostriction, and just experimental data). The authors of the review adhere to the point of view that a unified theory of EPE can hardly be created in the near future only on the basis of experimental data; intensive research based on modeling is required. 

The authors of another review on the physical nature of electroplasticity [44], based on taking into account grain boundaries as defects, the finite element method, and experimental data, believe that the charge imbalance near defects sharply weakens the atomic bond under the action of an electric current. Therefore, microscale modeling of the temperature near the defects by the FE method is necessary. This approach was confirmed by measuring the elastic modulus, reflecting the bond strength of atoms.

Mathematical models often describe the behavior of a material during deformation with an electric current based only on the heating effect. The range of works considering other factors is quite limited.

In [80], the temperature distribution modeled during the tension of the AZ31 alloy using an electric current agrees well with experimental results. It was assumed that all the applied electrical energy contributes to heating. However, it is noted that, along with volume heating, the so-called microheating takes place. It depends on the inhomogeneity of the structure. Microheating is difficult to estimate, but, in combination with macroheating, this is enough to explain the EPE. Despite the accuracy of temperature determination, the authors [81,82] failed to accurately simulate the stress during deformation with current without the athermal component of the EPE. In [83], using a model of crystal plasticity, it was shown that, apparently, magnetic depinning is the most reasonable mechanism for explaining electroplasticity.

In [84], a common model including the effect of the strain rate was used. They found a correlation between the sample temperature and the applied current density in the form of T ≈ j^2^. A model based on localized Joule heating and the Hall–Petch ratio is presented in [85]. They noticed that the Hall–Petch effect, when using tension with current, was less compared to heating in a furnace. Their study concluded that the mechanism underlying the observed effects cannot consist solely of thermal softening. In a similar work [86], the effect of grain size and sample size on the behavior during thermal softening of AZ31 magnesium alloy was investigated. The authors proposed a semiempirical model that could successfully predict the relationship between the softening parameter and the current density for five technical metals. The proposed model could also accurately estimate the critical current density at which EPE is observed. A comprehensive review of electroplastic models based on Joule heating can be found in [87].

In [88], the authors think that heating is insufficient to explain the causes of EPE and include the effect of electron wind to explain the phenomenon of electroplasticity in the AZ31 alloy. They analytically assessed and showed that Joule heating prevails in the electroplastic effect, and the effect of the electron wind is relatively small. In [89], a common model was proposed using an internal state variable linking a thermoelastic/viscoplastic damage model and electromagnetic phenomena. In [66], a model based on the calculation of changes in dislocation density is proposed. In [90,91], a similar attempt was made using the dislocation density in the model for heat-resistant alloys, and the effect of grain size was also taken into account. The proposed model makes it possible to accurately predict the drawing force required.

**Figure 15:**
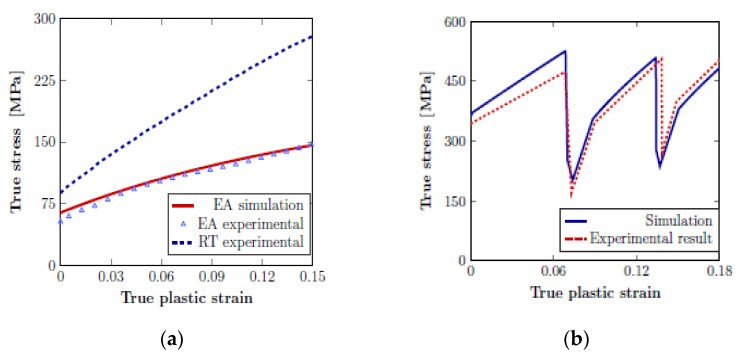
Finite element simulation result of the electric assisted (EA) compression test with corresponding experimental data: (**a**) at continuous current; (**b**) at pulsed current conditions [92].

The key observation during deformation by electric current is a steady drop in the flow stress and the effect of thermal softening depending on the stress–strain ratio and the restoration of properties when the current is switched off. These points are discussed in [44,92]. In [92], a model of EPE under tension based on the finite element method using the ABAQUS^®^ program is described. It is shown that it is pertinent to model the electroplastic phenomenon as the superposition of rate-dependent and rate-independent components of flow-stress evolution. The important characteristics of electric-assisted deformation reported in the literature, such as an instantaneous stress drop, recovery during the removal of electric current, and long-range softening, are predicted successfully using the dislocation density-based constitutive model. The implemented model is shown to simulate the experimental data very well in both continuous and pulsed current conditions (Figure 15).

In [93], the authors claim that heating and the mechanisms of the “electron wind” are sufficient to explain the stress decrease in titanium and offer their own explanation (scattering of dislocations by thermal phonons and electrons).

Thus, over the past 10 years, a significant number of papers on EPE modeling have appeared. This is due to the desire of researchers to better understand the nature of the effects since the direct staging of experiments is sometimes difficult due to many mechanisms being involved in EPE (Joule heating, electron wind, pinch, skin effects, etc.). At the same time, a distinctive feature of the studies in recent years is the emphasis on modeling the athermal mechanisms of EPE. However, a significant disadvantage of all modeling work is the practical lack of approaches that determine these effects in fine-grained and nanostructures, which today is one of the most important areas of research in physical materials science. The creation of EPE models at the nanoscale or atomic levels is relevant since the main proposed mechanisms of nonthermal nature take place at this scale. It should also be noted that, despite the variety of works on EPE modeling, there is a lack of views on the nature of the accompanying phenomena.

## 7. Application of EPE

Let us consider several areas of potential application of EPE. The main direction for use can be considered as a combination of electric pulsed current and metal forming for manufacturing of semifinished products of different shapes (rod, wire, sheet, and foil) from structural metals and alloys [20]. 

The method of electroplastic cold rolling has become the most widespread. It was applied to materials based on titanium [28,45], aluminum [94,95,96], magnesium [97], TiNi shape-memory alloys [67,98], and steels [99]. The benefit of combining external influences was observed in an increase in deformability, reduction of force, and improvement of productivity (by reducing the number of technological operations). These effects are not achieved in noncurrent processing. In some cases, the electroplastic rolling improved the surface quality by reducing roughness and increasing the mechanical properties. It was shown that the use of EPE was especially important for hard-to-form or brittle metals, for example, for tungsten and its alloys [20,100].

Drawing with current has demonstrated effects similar to rolling with current [20,101]. Electroplastic deformation has a significant impact on the physical and mechanical characteristics of the product. Thus, the elongation increases, the number of kinks increases, and the time resistance slightly decreases. Electroplastic drawing leads to a decrease in resistivity as well. This fact opens up certain possibilities in simplifying the technological process of manufacturing aluminum wire with improved characteristics by replacing conventional drawing by electroplastic drawing. This change will allow the exclusion of energy-consuming annealing operations from the technological cycle. In addition, the proposed drawing technology, in comparison with the usual one, reduces the deformation forces. It leads not only to a reduction in energy costs but also increases the life of the mill, including the wear resistance of the deformation parts of the drawing. 

A potentially promising direction for using EPE could be the technology of obtaining microwires with a giant magnetic impedance [102,103], where the combination of the thermal effect of current and plastic deformation is an effective technological way.

It is known that pulsed current is used in higher speed processes, such as stamping and bending [104], when not only deformability increases but also springiness decreases [23].

**Figure 16:**
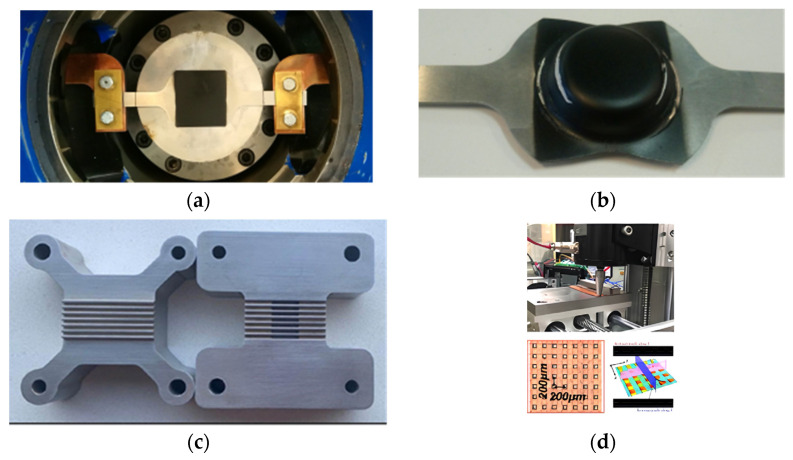
The setup of the deep drawing sample (**a**); example draw piece of 5754-H22 AA (**b**) [110]; precision stamping of microchannel features (**c**) and microimprinting of large-area features (**d**) [111].

In addition to metal forming processing or turning, other original technological solutions using pulsed current are known. These include cutting [105,106,107], pressure welding of metals [108], equal-channel angular pressing [109], and the possibility of structural refinement [3]. We also note the recently published review of the directions of EPE application [6] and the specific application of deep drawing technology using electric current on the example of an Al–Mg alloy (Figure 16) [110]. Figure 16 also shows examples of the possible use of pulsed current in microforming processes to produce elements with microsurface features [111].

Currently, several dozen machine tools and metalworking machines have been created on the basis of EPE in different countries. One example is a mill designed and created in Russia by the order of South Korea for rolling a strip of stainless steel with a cross section of 2 × 100 mm^2^ to a thickness of 0.3 mm at a speed of 0.5 m/s without intermediate annealing [112]. 

Summarizing the above-mentioned applications in industry, we can note a wide range of studies and potential methods aimed at intensifying technologies and improving the quality of structural materials. However, there are relatively few really working technologies that are commercially in demand. This is due to the limitation of devices (generators) in terms of the maximum pulse-current density, and, consequently, the cross section of the processed products, as well as the forced low deformation rate in production processes.

## 8. Conclusions

This review has been prepared for a better understanding of the progress that has been made in the field for the knowledge, understanding, and application of the phenomenon of electroplasticity in different materials. Over the past few years, it has become clear that the interest of scientists in the manifestation of electroplasticity has significantly increased. The number of published articles in the world on this topic is growing and contains more than a hundred per year.

Among the studied external and internal factors that strongly influence the effects of current in conductive materials, new ones have appeared; for example, the pulse-current duty cycle and grain size in a wide range, which expand our understanding of the electroplastic effect’s nature. Studies of the EPE in materials with shape memory and the use of current modes that stimulate the relaxation phenomena without significant heating are of particular interest.

The observed structural features during deformation with current are local in nature. There are small differences between microstructure after deformation with and without current. This fact leads to additional interest from researchers but also complicates the task of understanding and explaining the electroplastic effect’s nature, which remains open to this day.

The simultaneous effect of deformation and current can have a significant impact on the technological properties (increase in deformability) and also can be one of the ways to control the physical, mechanical, and functional properties (electrical, shape-memory properties, etc.) of alloys of different natures.

Due to the variety of emerging and interacting phenomena accompanying the combination of deformation with current, the necessity of creating a unified theory of electroplasticity becomes clearer. In this regard, modeling, especially at the micro-, nano-, and atomic levels, is an extremely important trend for fundamental and applied EPE research.

As for the use of electroplasticity, we can note a relatively small number of such examples in real industry, compared with research. In most cases, this is due to the insufficiently high technical characteristics of pulse generators and low deformation rate.

## Data Availability

No new data were created in this study. Data sharing is not applicable to this article. The data presented in this review are available in the references.
